# Learning from Hindsight: Examining Autonomic, Inflammatory, and Endocrine Stress Biomarkers and Mental Health in Healthy Terrorism Survivors Many Years Later

**DOI:** 10.1017/S1049023X24000360

**Published:** 2024-10

**Authors:** Phebe Tucker, Betty Pfefferbaum, Carol S. North, Yan Daniel Zhao, Pascal Nitiema, Rachel Zettl, Haekyung Jeon-Slaughter

**Affiliations:** 1. Emeritus and Volunteer Faculty, Department of Psychiatry and Behavioral Sciences, The University of Oklahoma Health Sciences Center, Oklahoma City, Oklahoma USA; 2. Adjunct Professor of Psychiatry (Volunteer), The University of Texas Southwestern Medical Center, Dallas, Texas USA; 3. Associate Dean for Research, Presidential Professor, College of Public Health, Biostatistics, and Epidemiology, The University of Oklahoma Health Sciences Center, Oklahoma City, Oklahoma USA; 4. Assistant Professor, Department of Information Systems, W. P. Carey School of Business, Arizona State University, Tempe, Arizona USA; 5. Assistant Professor, Child and Adolescent Psychiatry, Department of Psychiatry and Behavioral Sciences, University of Oklahoma Health Sciences Center and OU Health, Oklahoma City, Oklahoma USA; 6. Assistant Professor, Department of Internal Medicine, UT Southwestern Medical Center, Statistician/Section Chief of Analytics, Research Service, VA North Texas HCS, Dallas, Texas USA

**Keywords:** biological stress markers, cortisol, Interleukin 1-β, Interleukin 2-R, terrorism

## Abstract

**Introduction:**

Terrorism and trauma survivors often experience changes in biomarkers of autonomic, inflammatory and hypothalamic-pituitary-adrenal (HPA) axis assessed at various times. Research suggests interactions of these systems in chronic stress.

**Study Objective:**

This unprecedented retrospective study explores long-term stress biomarkers in three systems in terrorism survivors.

**Methods:**

Sixty healthy, direct terrorism survivors were compared to non-exposed community members for cardiovascular reactivity to a trauma script, morning salivary cortisol, interleukin 1-β (IL-1β), and interleukin 2-R (IL-2R). Survivors’ biomarkers were correlated with psychiatric symptoms and diagnoses and reported functioning and well-being seven years after the Oklahoma City (OKC) bombing.

Main outcome measures were the Diagnostic Interview Schedule (DIS) Disaster Supplement for Diagnostic and Statistical Manual of Mental Disorders, Fourth Edition, Text Revision (DSM-IV-TR) diagnoses, Impact of Events Scale-Revised (IES-R), Beck Depression Inventory-II (BDI-II), Distress and Functioning Scale (DAF), and General Physical Well-Being Scale.

**Results:**

Survivors had higher inflammatory IL-1β, lower anti-inflammatory IL-2R, lower cortisol, higher resting diastolic blood pressure (BP), and less cardiovascular reactivity to a trauma script than comparisons. Survivors’ mean posttraumatic stress (PTS) symptom levels did not differ from comparisons, but survivors reported worse well-being. None of survivors’ biomarkers correlated with PTS or depressive symptoms or diagnoses or reported functioning.

**Conclusions:**

Alterations of biological stress measures in cardiovascular, inflammatory, and cortisol systems coexisted as an apparent generalized long-term response to terrorism rather than related to specific gauges of mental health. Potential interactions of biomarkers long after trauma exposure is discussed considering relevant research. Longer-term follow-up could determine whether biomarkers continue to differ or correlate with subjective measures, or if they accompany health problems over time. Given recent international terrorism, understanding long-term sequelae among direct survivors is increasingly relevant.

## Introduction

### Background

Research has identified numerous biological markers associated with terrorism and traumatic events in general. Specific studies of autonomic nervous system biomarkers in terrorism include a prospective study of Israeli terrorist attack victims with early posttraumatic stress disorder (PTSD) symptoms compared to motor vehicle victims at one week and four months after trauma; terrorist survivors had more PTSD symptoms and higher heart rates (HRs) regardless of PTSD symptoms at both times.^1^ A longer-term study drawing from the same Oklahoma City (OKC) bombing (Oklahoma USA; 1995) sample as the current study identified greater autonomic reactivity in direct survivors of terrorism seven years later, independent of PTSD diagnosis.^2^ Of note, spouses of OKC bombing survivors had higher autonomic reactivity and higher afternoon salivary cortisol than community controls seven years post-disaster,^3^ and child relatives of victims had long-term physiological reactivity and hypothalamic-pituitary-adrenal (HPA) axis effects after seven years.^4^ Sarin gas victims in the Tokyo subway attack (Japan; 1995) with PTSD had elevated skin conductance.^5^ Considering other types of trauma, relocated Hurricane Katrina (Gulf Coast USA; 2005) survivors assessed 19 months after hurricane exposure had at baseline higher resting HR, lower parasympathetic baseline heart rate variability (HRV), and higher sympathovagal HRV than controls. Survivors with depression and with depression plus PTSD had flattened parasympathetic responsiveness to trauma cues.^6,7^

Studies of cortisol secretion have varied in different traumatized populations at various times. Both high and low cortisol levels were found in combat veterans with PTSD.^8^ Cortisol assessments of civilian survivors of acute trauma treated in an emergency room showed that PTSD and non-PTSD survivors had similar plasma cortisol levels at one and five months, but levels decreased over time.^9^ However, among soldiers assessed at three times, a negative correlation between morning salivary cortisol and posttraumatic stress (PTS) symptoms was found five days after exposure to trauma, and a positive correlation between cortisol levels and PTS symptoms was found two and nine months later, indicating that HPA axis changes may shift over time.^10^ Starting three months after the World Trade Center (WTC; New York USA) attacks on September 11, 2001, treatment-seeking survivors with PTSD had lower urinary cortisol excretion than non-PTSD survivors, with levels negatively associated with PTSD symptoms.^11^ Another study assessed highly-exposed September 11 survivors, at seven and 18 months post-disaster, finding elevated cortisol in men associated with more severe re-experiencing symptoms and fewer avoidance symptoms.^12^ A long-term study of direct survivors of terrorism, drawing from the current sample, identified higher salivary cortisol levels among survivors with PTSD compared to non-PTSD survivors and comparison participants seven years post-disaster, whereas autonomic reactivity was a generalized trauma response in survivors.^13^

Cytokines, modulators of the immune response that are produced by immune cells, have reciprocal influences on cortisol and cortisol releasing hormone. Studies at various times after terrorism exposure have found several cytokines to differ in those with and without trauma exposure or PTSD. Interleuin-2 or interleukin 2-R (IL-2R) as assessed in earlier studies, a T-cell growth factor, is involved with cell-mediated immunity, a system protecting against viruses, fungi, and other foreign pathogens. Interleukin-2 affects differentiation of helper T-cells modulating cytokine receptor responsiveness and signaling of several other cytokines.^14^ Earthquake survivors in northern China with or without PTSD had significantly lower serum interleukin-2 levels and more severe psychological symptoms than non-exposed comparison participants when assessed three months post-disaster.^15^ Considering PTSD, serum IL-2R was found to be lower in chronic PTSD of mixed trauma type, and selective serotonin reuptake inhibitor (SSRI) treatment normalized this cytokine.^16^ Serum IL-2R was no different in chronic combat-related PTSD compared with normal comparison participants.^17^

Interleukin 1-β (IL-1β) is a pro-inflammatory cytokine that may be inhibited by cortisol. Among military personnel, a review article identified neuroendocrine (including cortisol), inflammatory (including IL-1β) growth and anabolic biomarkers as associated with stress and stress adaptation.^18^ Among individuals exposed to various types of traumatic events, individuals with PTSD related to a deadly earthquake had higher IL-1β than survivors without PTSD but no differences in IL-2 when assessed 5.5 years later.^19^ Interleukin-1β was higher in Israeli combat veterans with PTS symptoms for a mean of 7.5 years compared with healthy volunteers.^17^ Another study found that participants with chronic PTSD from diverse trauma had significantly greater PTSD, depression, and IL-1β and lower IL-2R levels than comparison participants, with no group differences found for morning or afternoon cortisol levels. Treatment with SSRI was associated with lowered PTSD, depression, and IL-1β levels and increased IL-2R for all groups relative to levels of comparison participants.^16^ Women who were sexually assaulted were followed-up at six months and one year; while there were no baseline differences in IL-1β or cortisol compared to controls, after treatment with interpersonal psychotherapy or SSRI, victims’ IL-1β and cortisol were higher than in comparison participants despite improvement in depression and PTSD symptoms. Authors suggested that immunological symptoms may persist in the long term despite improvement in emotional symptoms.^20^ Longer-range assessments of young adults who were traumatized as children showed increased IL-1β levels were higher with severity of trauma.^21^

Recent research has explored the complex relations of the central nervous system (CNS), immune system (IS), and endocrine system (ES) in the chronic stress response. Zefferino examined the interaction of cortisol, norepinephrine (NE), IL-1β, and melatonin proposing that IL-1β is the main chronic stress biomarker, signaling NE and the ES (cortisol and melatonin). Moreover, with cortisol resistance, IS, ES, and CNS networks are unbalanced.^22^Among soldiers in stressful training for up to 12 weeks in duration, Beckner’s review article examined stress biomarkers across several systems, including ES (cortisol), NE, and inflammatory (including IL-1β) and anabolic, in stress adaptations and resilience. She suggested an interaction and balance in stress adaptation.^18^

Several studies have assessed emotional and psychosocial functioning as well as psychopathology of direct OKC bombing survivors over time. In their landmark study of OKC bombing survivors, North and colleagues^23^ found that disaster-related PTSD had developed in 34% of survivors by six months after the disaster, and nearly one-half had a post-disaster psychiatric disorder. By seven years post-disaster,^24^ 26% were experiencing current PTSD and 10% had current major depression, but delayed-onset PTSD and new-onset post-disaster alcohol use disorders did not occur. The sample for the current study was obtained from the original North, et al OKC bombing survivor study sample.^23^ Prior studies from this sample have examined a limited set of biomarkers.^2,13^ The current study builds on these studies by reporting as-yet unpublished data on interleukins, representing the IS, and relating it to previously assessed cardiovascular reactivity and cortisol.

### Objective

This study provides an unprecedented long-term, retrospective assessment of several representative biological markers in three distinct systems--cardiovascular reactivity (related to CNS NE), morning cortisol (ES), and IL-1β (IS) and IL-2R (IS) seven years after direct exposure to the OKC terrorist bombing. Subjective and objective stress measures are compared with those of non-exposed community comparison participants and their associations are examined. While studies show that subjective and biological stress measures may attenuate with time, understanding their relationships as long as seven years later may help identify long-range physical as well as mental health issues. Research assessing multiple long-term biological markers in terrorist survivors from the same event is lacking. Conducting such research is challenging, and this study was possible due to the early commitment of survivors to help promote better understanding of terrorism’s adverse effects on mind and body.

## Methods

### Participants

Of 113 adult direct survivors of the OKC bombing who participated in a follow-up study by North and colleagues 6.5-to-seven years later,^24^ study participants for a biological assessment were enlisted by letter and/or telephone call starting late October 2001. North’s sample had been drawn from her earlier six-months post-disaster study of 182 direct survivors, 87% injured,^25^ with the sample randomly selected from an Oklahoma State Department of Health (OSDH; OKC, Oklahoma USA) registry of 1,092 survivors collected as a public record of directly exposed individuals. Non-exposed community control participants were recruited through flyers or word of mouth. All participants were free of psychiatric and other medications or medical conditions that could affect biological measures. Of the 71 survivors who underwent biological assessments, all assessments were completed by 60 adult survivors and 23 controls who had not been directly or indirectly (through relative or acquaintance) exposed to the bombing. Written informed consent was obtained from both Institutional Review Boards of the University of Oklahoma Health Sciences Center (#09031; OKC, Oklahoma USA) and Washington University School of Medicine (#88-0832 and #00-0922; St. Louis, Missouri USA). All participants were paid US$150 for their participation. The authors assert that all procedures contributing to this work comply with the ethical standards of the relevant national and institutional committees on human experimentation and with the Helsinki Declaration of 1975, as revised in 2008.

### Procedures

*Biological Measures—*Lab technicians performing all biological assessments and assays were blind to participants’ identity. Survivors and controls underwent all procedures.

Salivary cortisol samples were collected in the morning immediately after informed consent was obtained to avoid having levels affected by other procedures such as blood draws and physiologic and psychometric assessments. Participants were instructed to abstain from caffeine and nicotine prior to assessments. Further methods for obtaining and processing samples are provided in previous publications.^13^

Interleukins 1β and 2R were then obtained from fasting blood samples also prior to the physiologic and psychometric assessments to avoid the possibility they might trigger stress affecting interleukin levels. Specific details on assays of cytokines (Mayo Clinical Trial Services; Rochester, Minnesota USA) are described in an earlier publication.^26^

Physiologic assessments were performed next, measuring HR and blood pressure (BP) reactivity to a trauma interview. Procedures are described in an earlier publication.^2^ The HR was recorded via standard electrocardiogram (ECG) Lead II Placement (Lablink Modular Instrument System, Biopac Systems, Inc.; Goleta, California USA). Systolic, diastolic, and mean arterial BP were measured every 30 seconds by a Critikon Dinamap Vital Signs Monitor (1846 SX, GE Healthcare; Milwaukee, Wisconsin USA), interfaced with a Compaq computer (Hewlett-Packard; Palo Alto, California USA). Both HR and BP reactivity were measured in three phases of four minutes each before, during, and after a semi-structured interview about the bombing that asked participants to describe how they felt after experiencing or learning about the bombing, if they knew anyone injured or killed, and if there was anything that jumped into mind when thinking about the bombing. A difference score was calculated for the baseline pre-test measures and the measures during the interview; this represented participants’ physiological reactivity to trauma cues.

*Psychometric Measures—*The Diagnostic Interview Schedule (DIS) Disaster Supplement assessed diagnoses, scored according to Diagnostic and Statistical Manual of Mental Disorders, Fourth Edition, Text Revision (DSM-IV-TR), for the past month for the seven-year study by North and colleagues.^24^ In this study, PTSD, major depression, and presence of any psychiatric diagnosis were included in analyses.

Items from the 22-item self-report measure, the Impact of Events Scale-Revised (IES-R; for DSM-IV-TR), assessed subjective distress caused by traumatic events. The IES-R is a revised version of the original 15-item IES^27^ with seven additional items related to the PTS hyperarousal symptoms.^28^ To maintain consistency with prior studies, in the current study, each item was rated on a four-point scale (one = not at all; two = rarely; three = sometimes; and four = often), with possible scores ranging from 22 to 88. This scale is referred to as IMPOK (for Impact OKC) in this study.

All participants were administered the Beck Depression Inventory-II (BDI-II).^29,30^ This self-report inventory consists of 21 items, each scored from zero to three, with three representing the most severe level of the symptom in question. The BDI-II total scores range from zero to 63. A score of zero-to-13 is considered minimal range, 14-19 is mild, 20-28 is moderate, and 29-63 is severe. In non-clinical populations, scores above 20 indicate depression.

Further measures include a Distress and Functioning Scale (DAF), which has 28 questions about difficulties in the past week with emotional distress; functioning at work, home, and school; being upset; having difficulty enjoying or relaxing; or having problems in interacting with others. Items were scored from zero (none) to four (almost always). A general physical well-being scale contained two questions. The first queried whether the participant was bothered by illness, bodily disorders, pains, or fears about health on a scale of one (all the time) to six (none of the time), with lower scores indicating worse health. The second question had participants choose a number indicating how worried they were about health, rated from zero (not at all concerned) to ten (very concerned).

### Data Analyses

Subjective measures of BDI-II, IMPOK (revised IES-R), DAF, and general well-being were compared for survivors and comparison participants. Similarly, biological stress markers of baseline (pre) and reactivity (diff) HR, systolic BP, and diastolic BP were also compared for survivors and controls, as were levels of morning salivary cortisol, IL-1β, and IL-2R. Multivariable regression models compared means for survivors and controls.

Univariate associations and Spearman correlation coefficients assessed relationships between subjective measure of depression and biological stress measures. For univariate association, a cutoff score of 19 was used for BDI-II to sort out those with scores indicating the presence of depression. For univariate associations, significance was defined as non-parametric P values of P <.05.

Spearman correlations compared continuous BDI-II, IMPOK (revised IES), DAF, and general physical well-being variables with biomarkers.

Correlations of biomarkers with diagnoses of PTSD, major depression, or any mental disorder in the past month were calculated by the Kruskal-Wallis test for non-parametric P values, with significance at P <.05.

## Results

Socio-demographics of participants were: survivors were 54.7% male, 86.8% Caucasian, and mean age of 47 years. Comparison participants were 46.1% male, 79.5% Caucasian, and mean age of 42 years.

### Group Differences between Survivors and Controls

*Mental Health Ratings*—Comparing median BDI and IMPOK (revised IES-R) between the groups, survivors’ levels were nearly significantly higher than the comparison group (P = .051 versus P = .055). The first general physical wellness question showed that survivors felt significantly less well than comparisons (4.3 versus 4.93; P = .018). There were no significant group differences on other subjective rating scales, the DAF, and the second general physical wellbeing question. For IMPOK, the survivor group’s average rating was 1.89, below the “rarely” option (Table 1). Survivors’ mean BDI scores were below clinically relevant levels. The DIS and DSM-IV-TR diagnoses in the past month for survivors was 17.6% (N = 19) for PTSD, 3.7% (N = 4) for depression, and 13.8% (N = 15) for any psychiatric diagnosis.

**Table 1.** Comparison of Subjective Ratings for Survivors and Controls in Past Month

	IMPOK (Revised IES)	BDI-II	Distress & Functioning (DAF)	General Well-Being 1	General Well-Being 2
*Survivors*					
Mean	41.55	8.25	66.69	4.3	4.29
95% CI	38.16-44.94	7.36-10.14	60.13-73.24	3.99-4.61	3.74-4.83
*Controls*					
Mean	35.81	5.96	58.22	4.93	3.85
95% CI	29.28-42.34	1.88-10.04	46.14-70.31	4.32-5.54	2.72-4.98
Survivors vs Controls, Non-Parametric P Value	.055	.051	.197	**.018** ^a^	.396

Note: General Well-Being 1-Was person bothered by illness, bodily disorders, pains, or health fears? General Well-Being 2-How worried was person about health?

Abbreviations: IMPOK, revised Impact of Events Scale (IES)-Revised; BDI-II, Beck Depression Inventory-II.

a Significant at P <.05.

*Interleukins and Cortisol*—Multivariable linear model assessing the association between immunological markers for OKC bombing exposure adjusting for gender, age, ethnicity, and psychiatric diagnosis found that the average IL-1β was higher in survivors (mean = 116.76) compared to comparison participants (mean = 98.3); P <.001. The average IL-2R was lower in survivors (mean = 435.7) compared to comparison participants (mean = 503.17); P = .021). Mean morning cortisol for survivors was significantly lower than comparisons (0.27 versus 0.87; P = .022; Table 2).

**Table 2.** Comparison of Biological Stress Markers for Survivors and Controls

	Pre- HR	Pre- Sys. BP	Pre- Dias. BP	Diff HR	Diff Sys. BP	Diff Dias. BP	IL 1β	IL 2-R	AM Cortisol
*Survivors*									
Mean	70.42	121.01	75.56	5.45	14.38	8.28	116.76	435.7	0.27
95% CI	67.46-73.38	116.49-125.52	72.91-78.21	3.93-6.98	11.85-16.92	6.98-9.57	111.83-121.69	416.93-454.48	0.24-0.30
*Controls*									
Mean	68.41	115.35	68.26	8.91	20.13	11.26	98.3	503.17	0.87
95% CI	63.96-72.86	109.49-121.21	65.31-71.21	6.36-11.47	15.67-24.59	9.09-13.43	87.81-108.79	454.76-551.57	−3.31-5.05
Survivors vs Controls, Non-Parametric P Value	.326	.265	**.003** ^**a**^	**.017** ^**a**^	**.036** ^**a**^	**.028** ^**a**^	**<.001** ^**a**^	**.021** ^**a**^	**.022** ^**a**^

Abbreviations: Pre-HR, pre-test heart rate; Pre-Sys BP, pre-test systolic blood pressure; Pre-Dias BP, pre-test diastolic blood pressure; Diff HR, testing minus pre-test heart rate; Diff Sys BP, testing minus pre-test systolic blood pressure; Diff Dias BP, testing minus pre-test diastolic blood pressure; IL-1β, Interleukin 1β; IL 2-R, Interleukin 2-R; AM cortisol, morning salivary cortisol.

a Significant at P <.05.

*Cardiovascular Measures*—For cardiovascular measures, survivors had significantly higher levels of baseline (pre) diastolic BP than comparisons, and significantly lower reactivity (difference score, testing minus baseline) to trauma cues for HR and systolic and diastolic BP (Table 2).

### Correlations between Subjective Scales and Biological Stress Markers

Univariate association revealed no significant results. Using a cut-off score of 19 for depression on BDI-II, there were no significant associations of BDI scores for any biological marker (morning cortisol: P = .379; resting HR: P = .224; resting systolic BP: P = .424; resting diastolic BP: P = .797; difference [diff] HR: P = .069; diff systolic bp: P = .141; diastolic bp: P = .156; IL-1β: P = .241; and IL-2R: P = .338).

Spearman correlations comparing continuous BDI-II (Table 3), IMPOK (revised IES-R; Table 4), DAF, and General Wellness-1 variables with biomarkers showed no significant correlations for any of the biological stress measures.

**Table 3.** Spearman Correlations for BDI Compared with all Biological Markers

Spearman Correlation Coefficients: BDI Prob > |r| under H0: Rho = 0 Number of Observations									
	AMMEAN	PREHR	PRESYS	PREDIA	DIFFHR	DIFFSYS	DIFFDIA	IL1B	IL2R
**SC** **P Value** **N**	0.00675 .9493 91	−0.11612 .4269 49	−0.03549 .8087 49	−0.05190 .7232 49	−0.15702 .2813 49	−0.14472 .3211 49	−0.17298 .2346 49	−0.09065 .5356 49	0.01487 .9192 49

Abbreviations: SC, Spearman Correlation with BDI; BDI, Beck Depression Inventory-II; AMMEAN, morning salivary cortisol; PREHR, pre-test heart rate; PRESYS, pre-test systolic blood pressure; PREDIA, pre-test diastolic blood pressure; DFFHR, test minus pre-test heart rate; DIFFSYS, test minus pre-test systolic blood pressure; DIFFDIA, test minus pre-test diastolic blood pressure; IL1B, Interleukin 1-β; IL2R, Interleukin 2-R.

**Table 4.** Spearman Correlations for IES-R (IMPOK) Compared with All Biological Measures

Spearman Correlation Coefficients: IMPOK (IES-R) Prob > |r| under H0: Rho = 0 Number of Observations									
	AMMEAN	PREHR	PRESYS	PREDIA	DIFFHR	DIFFSYS	DIFFDIA	IL1B	IL2R
**SC** **P Value** **N**	−0.05098 .6313 91	0.08687 .5529 49	−0.05927 .6858 49	0.04638 .7517 49	0.09639 .5100 49	0.11164 .4451 49	0.10510 .4723 49	−0.05260 .7197 49	0.18347 .2070 49

Abbreviations: SC, Spearman Correlation Coefficient; IMPOK, Impact OKC for Impact of Events Scale-Revised (IES-R); AMMEAN, morning salivary cortisol; PREHR, pre-test heart rate; PRESYS, pre-test systolic blood pressure; PREDIA, pre-test diastolic blood pressure; DFFHR, test minus pre-test heart rate; DIFFSYS, test minus pre-test systolic blood pressure; DIFFDIA, test minus pre-test diastolic blood pressure; IL1B, Interleukin 1-β; IL2R, Interleukin 2-R.

Kruskal-Wallis test showed no significant correlations between any biomarker for survivors with and without PTSD, depression, or any psychiatric diagnosis (Table 5).

**Table 5.** Comparison of Biological Stress Markers for Survivors With and Without Psychiatric Diagnoses

	Pre- HR	Pre- Sys. BP	Pre- Dias. BP	Diff HR	Diff Sys. BP	Diff Dias. BP	IL 1β	IL 2-R	AM Cortisol
PTSD Survivors									
Mean	70.71	121.53	75.73	5.52	13.98	8.22	116.52	432.81	0.26
95% CI	67.69-73.72	116.60-126.45	72.79-78.66	3.83-7.21	11.49-16.47	6.88-9.55	111.21-121.83	413.00-452.62	0.23-0.29
Non-PTSD Survivors									
Mean	68.26	117.07	72.61,	4.96	17.43	8.71	118.57	457.63	0.33
95% CI	53.77-82.74	103.54-130.60	67.62-80.95	1.40-8.51	3.51-31.35	2.61-14.82	101.03-136.11	382.79-532.47	0.22-0.45
Non-Parametric P Value	.572	.549	.872	.927	.712	.844	.791	.289	.264
Depressed Survivors									
Mean	70.88	121.35	75.59	5.49	14.31	8.29	116.46	436.5	0.28
95% CI	67.89-73.87	116.73-125.98	72.89-78.28	3.92-7.07	11.69-16.93	6.96-9.63	111.37-121.54	417.61-455.38	0.24-0.31
Non-Depressed Survivors									
Mean	57.15	111	74.75	4.35	16.5	7.75	125.5	412.65	0.12
95% CI	33.64-80.66	−3.36-225.36	−61.84-211.34	−0.10-8.80	−2.56-35.56	−14.49-29.99	122.96-128.04	−745.52-1570.82	−0.09-0.33
Non-Parametric P Value	.067	.333	.837	.934	.523	.967	.410	.757	.064
Survivors w/Any Psychiatric Dx									
Mean	71.1	121.55	75.64	5.52	13.8	8.15	116.49	433.18	0.26
95% CI	68.10-74.11	116.62-126.47	72.72-78.56	3.83-7.21	11.31-16.30	6.82-9.49	111.19-121.80	413.25-453.10	0.23-0.29
Survivors w/Out Psychiatric Dx									
Mean	65.26	116.93	74.93	4.96	18.79	9.21	118.79	454.83	0.33
95% CI	51.35-79.17	103.44-130.42	67.47-82.38	1.40-8.51	5.35-32.22	3.21-15.22	101.18-136.40	382.16-527.49	0.18-0.49
Non-Parametric P Value	.153	.519	.972	.927	.294	.588	.738	.345	.585

Note: Kruskal-Wallis test, significant at P <.05.

Abbreviations: PTSD, posttraumatic stress disorder; Pre-HR, pre-test heart rate; Pre-Sys. BP, pre-test systolic blood pressure; Pre-Dias. BP, pre-test diastolic blood pressure; Diff HR, testing minus pre-test heart rate; Diff Sys. BP, testing minus pre-test systolic blood pressure; Diff Dias. BP, testing minus pre-test diastolic blood pressure; IL-1β, Interleukin 1β; IL 2-R, Interleukin 2-R.

## Discussion

Prior studies have assessed biological stress measures and psychological symptoms and diagnoses after exposure to terrorism and trauma over time, ranging from shortly after the event to several years later. However, none were identified that assessed terrorism survivors’ biological stress measures in all three systems of ES (cortisol), IS (IL-1β and IL-2R), and CNS norepinephrine (NE - cardiovascular reactivity). In this study, among physically healthy and highly exposed direct survivors of the same terrorist event, multiple long-range biological stress systems were retrospectively assessed and compared to psychological stress symptoms and measures of comparison participants. In assessing the relationship of these subjective and objective measures, an attempt was made to determine whether they are consistent in reflecting enduring responses to severe trauma that might impact health and mental health over time.

Survivors’ PTSD and depression symptoms did not differ from those of comparison participants. While symptom levels for this group were not available for shortly after the bombing, an early study of survivors from the same state health department registry group six months after the bombing found that 45% had a post-disaster psychiatric disorder, with 34% having PTSD and 23% having major depression.^23^ Moreover, North’s seven-year post-disaster study of 113 direct survivors from the same OSDH’s original public registry found that 26% had active PTSD.^24^ This study assessed only medically healthy survivors who were not on cardiovascular or psychotropic medications that would confound the results of physiologic assessments, so these participants represent a unique, healthy subgroup. It is not known if participants had psychological or psychotropic treatments at any time prior to assessments. Of note, the only significantly different subjective finding was that survivors rated their general physical wellness lower than controls.

The current study is unique among studies of terrorism in assessing multiple long-range biological stress markers in survivors. Autonomic assessments found that for cardiovascular measures, survivors had significantly higher levels for baseline diastolic BP than comparison participants, but significantly lower reactivity to trauma cues (testing minus baseline scores) for all measures. This group’s cardiovascular response to trauma cues appears to be blunted after seven years. This may possibly be due to survivors’ higher baseline measures preventing a robust increase during the testing (trauma script) phase compared to comparison participants. Halligan, et al’s study of assault survivors found a reduced HR response to trauma reminders in those with PTSD compared to those without PTSD, despite greater distress. Authors noted that engagement in rumination predicted lower HR response.^31^ In the current study, none of these cardiovascular measures reflecting CNS (NE) correlated with symptoms of depression on BDI-II using univariate analysis with cutoff-scores for levels of depression, and no biomarkers correlated with continuous subjective variables, including BDI-II and IMPOK (IES-R); Table 3 and Table 4.

Assessments of morning salivary cortisol identified lower levels in survivors than comparison participants at seven years post-bombing. This is consistent with some other studies showing lower cortisol in the non-acute stage.^9,11^ These results differ from other studies that found higher cortisol after other types of trauma exposure.^10,12^ This diversity of findings reflects the complexity of cortisol studies whose results may vary with timing, methods, and trauma type. Similar to autonomic correlations, in the current study, cortisol levels, reflective of the endocrine system (ES), were not correlated with psychometric measures. This biomarker also appeared to be a lasting remnant of terrorism exposure. As cortisol can inhibit IL-1β, low cortisol levels in this study apparently did not inhibit this cytokine, which was elevated.

Interleukin-1β was significantly higher in survivors than comparison participants, which is compatible with studies showing this pro-inflammatory cytokine to be elevated at different times after trauma exposure.^15,17,19,20^ Zefferino^21^ postulated that IL-1β is the main chronic stress biomarker, signaling NE and the ES (cortisol and melatonin); however, it is not possible to reproduce this role in the current study. Of interest, D’Elia’s follow-up research showing IL-1β and cortisol levels to be higher one year after sexual assault, despite improvement in PTS and depression symptoms after treatment, is not unlike the current study showing higher IL-1β and other altered biological measures despite low symptom levels seven years after terrorism. In the current study, the only subjective measure differing from comparison participants for these terrorist survivors was the general wellness question, indicating that survivors rated their general health as worse. D’Elia suggested that immunological symptoms may persist in the long term despite improvement in emotional symptoms.^19^

Interleukin-2 or IL-2R, promoting cell-mediated immunity, was significantly lower in survivors than comparison participants in the current study as in some other studies.^15,16,19^ However, the current study diverges from the 2004 Tucker study that found selective serotonin reuptake inhibitor (SSRI) treatment increased IL-2R, while at the same time improving subjective measures of PTS and depression to levels of controls;^16^ in this former study, improvements in subjective measures were consistent with biological markers. The current study showed long-term lowering of IL-2R among healthy terrorism survivors (as well as cortisol levels) despite non-elevated mean levels of PTS and symptoms.

These findings of lasting stress biomarkers after direct terrorism exposure despite emotional healing or resilience raises the question of possible long-term effects on health in the survivors who were pre-selected to be medically healthy. Would these biomarkers leave them vulnerable to medical problems over time? Indeed, their only elevated subjective measure was their assessment of general health. A different subset of heavily impacted bombing survivors from the OKDH registry who were not selected for health reasons was assessed for subjective mental health and health 18.5 years later.^32^ This group reported receiving more care from physical, speech, respiratory, and occupational therapists than local controls. Much of this care from ancillary care services may have been a direct result of bombing injuries or exposure to dust or toxins. They also had more anxiety and depression, which was associated with heavy drinking, and 23.2% were found to have risk for PTSD.^33^ Knowing if any of this larger group had different biomarkers at seven years or later would help to better understand the role of biomarkers in various stress systems and on long-term health. Of interest, a review of health problems among survivors of the WTC disaster revealed major cardiovascular, pulmonary, and neurological health problems from WTC dust exposure. First responders were especially exposed to factors increasing their risks for age-related disease, including cardiovascular, pulmonary, and neurologic disease.^33^ Also, compared to the general New York population, first responders had a higher rate of all cancers, and higher rates of prostate cancer, thyroid cancer, and leukemia.^34^ The current study’s OKC survivors were exposed to dust and injuries.

## Limitations and Strengths

Limitations of this study include a failure to have periodic post-disaster assessments of biomarkers. Furthermore, as survivors were included only if free of medications or medical conditions that could confound biological assessments, selection bias is likely. These healthy survivors may have been more mentally healthy than the larger group. Nonetheless, several of their stress biomarkers were consistent with a lasting stress response. Not all available study participants were recruited into the study, so that non-participant error may have affected results. A lower number of comparison participants (n = 23) than survivors (n = 60) completed all three biological assessments in this study, due to fewer participants taking part in the interleukin blood draws. Effects of other trauma were not assessed. In addition, directly exposed survivors were assessed starting in October 2001; it is possible that the terrorist events of September 11 may have further sensitized their reactions through their contact with September 11 media coverage, as suggested by findings from OKC bombing survivors followed-up shortly after the September 11 attacks.^35,36^ However, given the occurrence of other terrorist events internationally, other studies of terrorism’s sequelae could also be affected by further acts of terrorism.

A strength is that all participants in this study were directly affected by the same terrorist event and agreed to be assessed long afterward, which is unusual and owes to their commitment to further research.

## Conclusion

Despite limitations, the current study reveals that medically healthy survivors’ biological stress measures in three systems differ from those of comparison participants, consistent with long-term consequences of trauma, while PTS and depressive symptoms show general self-reported emotional healing with mean levels below clinical concern. There was a disconnect between many of terrorism survivors’ long-term biological stress measures and emotional symptoms with a lack of correlations between them. Overall, the psychological mind appears to have released distress for many, while the brain and body biology has remembered long after. A follow-up study could determine an even longer-range trajectory of emotional and biological stress measures, and whether they are interconnected and associated with health problems with age. Biological stress markers remain to be examined on a very long-term basis. Terrorism continues internationally and warrants a better understanding of its sequelae.
